# Mutagenesis Targeting the S^153^ Residue Within the Transmembrane β-Hairpin of Mosquito-Larvicidal Mpp46Ab Affects Its Toxicity and the Synergistic Toxicity with Cry4Aa

**DOI:** 10.3390/biology14050489

**Published:** 2025-04-30

**Authors:** Tohru Hayakawa, Syun Yamaoka, Mami Asakura, Minako Hirano, Toru Ide

**Affiliations:** Graduate School of Interdisciplinary Science and Engineering in Health Systems, Okayama University, 3-1-1 Tsushima-naka, Kita-ku, Okayama 700-8530, Japan

**Keywords:** *Bacillus thuringiensis*, mosquito-larvicidal proteins, synergistic toxicity, *Culex pipiens* mosquito larvae, side-directed mutagenesis, electrophysiologic analysis

## Abstract

We constructed a library of Mpp46Ab mutants, in which S^153^ within the transmembrane β-hairpin was randomly replaced by other amino acids. Mutagenesis and subsequent primary screening yielded 10 different Mpp46Ab mutants in addition to the wild type. Remarkably, S^153^ was replaced with a more hydrophobic amino acid in most of the mutants, and the S153I mutant in particular exhibited significantly increased toxicity. Electrophysiologic analysis using artificial lipid bilayers revealed that the single-channel conductance and *P*_K_/*P*_Cl_ permeability ratio were significantly increased for S153I pores. This suggests that the formation of highly ion-permeable and highly cation-selective toxin pores increases the influx of cations and water into cells, thereby facilitating osmotic shock. In addition, the S153F, S153L, and S153I mutants exhibited significantly reduced synergistic toxicity with Cry4Aa. Electrophysiologic analysis showed that the S153F, S153L, and S153I mutants form toxin pores with a significantly reduced *P*_K_/*P*_Na_ permeability ratio and a significantly increased *P*_K_/*P*_Ca_ permeability ratio compared to wild-type pores. Thus, our results suggest that pore formation is central to the insecticidal activity of Mpp46Ab and that the ion permeability of toxin pores is a potential indicator correlated with both toxicity and synergistic toxicity with other toxins.

## Simple Summary

The mosquito-larvicidal activity of Mpp46Ab, a pore-forming toxin derived from *Bacillus thuringiensis* TK-E6, has been shown to correlate with the cation selectivity of the toxin pores. In particular, K^155^ within the transmembrane β-hairpin is considered a good target for the improvement of Mpp46Ab, as increased cation selectivity of the toxin pores and consequently increased mosquito-larvicidal activity were observed in K155E and K155I mutants. In this study, to investigate the importance of S^153^ adjacent to K^155^, we constructed a mutant library, in which S^153^ was randomly replaced by other residues. After mutagenesis and subsequent primary screening using *Culex pipiens* mosquito larvae, we obtained 10 different Mpp46Ab mutants in addition to the wild type. Interestingly, the S153I mutant exhibited significantly increased toxicity. In addition, the S153F, S153L, and S153I mutants exhibited significantly reduced synergistic toxicity with mosquito-larvicidal Cry4Aa. Electrophysiologic analysis of the mutants revealed an apparent change in the ion-permeability of the toxin pores, which correlated with toxicity. Our results suggest that pore formation is central to the insecticidal activity of Mpp46Ab and that the ion permeability of toxin pores is a potential indicator correlated with both toxicity and synergistic toxicity with other toxins.

## 1. Introduction

A Gram-positive soil bacterium, *Bacillus thuringiensis* is generally characterized as an insect pathogen because it produces a variety of insecticidal proteins, primarily as a major component of protein crystals. These insecticidal proteins are used worldwide as “environmentally friendly” bioinsecticides [1]. However, the use of *B. thuringiensis* insecticidal proteins is always associated with the risk of selecting insecticidal resistance in the larval population. To overcome this potential drawback, an effective strategy for increasing the toxicity of insecticidal proteins is needed.

Mpp46Ab, a toxin derived from *B. thuringiensis* TK-E6, has a relatively broad spectrum and exhibits selective cytotoxicity against human leukemic T cells and mosquito-larvicidal activity against *Culex pipiens* mosquito larvae [2,3]. Notably, the co-administration of Mpp46Ab with another mosquito-larvicidal toxin, Cry4Aa from *B. thuringiensis* subsp. *israelensis* (Bti), results in significant synergistic toxicity against *C. pipiens* mosquito larvae [3]. Therefore, Mpp46Ab is expected to be used not only as a novel mosquito-larvicide but also as an agent to enhance the insecticidal efficacy of other mosquito-larvicidal toxins.

Mpp46Ab shares significant homology (84% identity) with Mpp46Aa from *B. thuringiensis* A1547 [4]. The three-dimensional structural model of Mpp46Aa has been constructed and shown to be structurally similar to aerolysin-type β-pore forming toxins (β-PFTs) [5]. Based on its similarity to Mpp46Aa, Mpp46Ab is also assumed to be an aerolysin-type β-PFT; indeed, Mpp46Ab forms cation-selective pores in the artificial lipid bilayer [3,6]. Aerolysin-type β-PFTs generally contain a characteristic β-hairpin structure in the middle domain of the toxin molecule. This β-hairpin is thought to be the transmembrane domain of the toxin, which forms a transmembrane β-barrel after insertion into the target cell membrane [7,8]. The β-hairpin generally contains an alternating pattern of polar and hydrophobic amino acid residues, which are thought to be located in the hydrophilic lumen and lipid bilayer of the β-barrel, respectively [9]. A similar β-hairpin structure has been identified in other aerolysin-type β-PFTs, such as aerolysin [10], staphylococcal α-toxin [11], ε-toxin from *Clostridium perfringens* [12], and mosquito-larvicidal Bin toxin from *Lysinibacillus sphaericus* [13]. Mpp46Ab also contains a β-hairpin structure in the region spanning β8 and β9 (residues L^152^ through T^168^).

We have previously speculated that the toxicity of Mpp46Ab is closely related to the ion permeability of the toxin pores, as pore formation is thought to be central to its mode of action. We constructed a series of Mpp46Ab mutants targeting a charged amino acid residue, specifically K^155^ in the β-hairpin, and showed an apparent correlation between cation selectivity of the toxin pores and mosquito-larvicidal activity [6]. Thus, we tentatively concluded that ion permeability, particularly cation selectivity of the toxin pores, is an important determinant of the mosquito-larvicidal activity of Mpp46Ab and that the ion permeability of the toxin pores can be controlled by mutagenesis targeting the β-hairpin region.

In the present study, we constructed Mpp46Ab mutants, in which residue S^153^ was randomly replaced with other amino acids. Residue S^153^ is located within the β-hairpin and is expected to face the hydrophilic lumen of the transmembrane β-barrel, as is residue K^155^. Mpp46Ab mutants exhibiting significant toxicity were screened using a simplified bioassay with *C. pipiens* mosquito larvae, and the mutation in each clone was identified by DNA sequence analysis. We then investigated the effects of replacing Mpp46Ab residue S^153^ on the toxin’s mosquito-larvicidal activity and its synergistic toxicity with Cry4Aa. To clarify the relationship between toxin pore ion selectivity and associated insecticidal activity, the selected Mpp46Ab mutants were subjected to electrophysiologic measurements using artificial lipid bilayers.

## 2. Materials and Methods

### 2.1. Construction of a Random Mutant Library Targeting the S^153^ Residue

**Figure 1:**
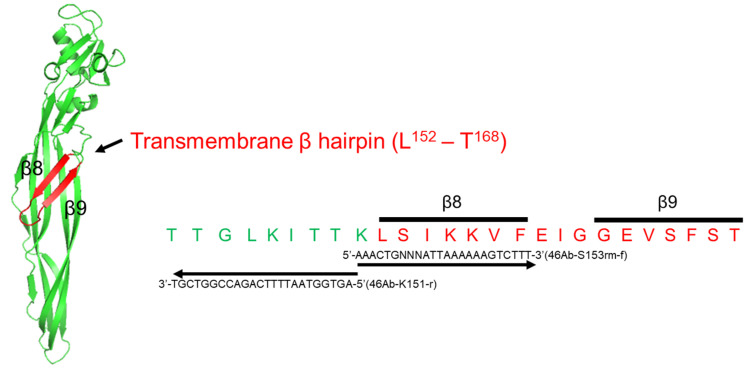
Three-dimensional structural model of Mpp46Ab and the transmembrane β-hairpin (β8-β9) region. The model was constructed using SWISS-MODEL [14,15] with the PDB code Mpp46Aa (2ztb). Residue S^153^ is located within the β8 region. The nucleotide sequences of the specific primer sets used to generate the random mutant library are shown.

Residue S^153^ of Mpp46Ab was randomly replaced by other amino acid residues, as previously described [6]. PCR was performed with a specific primer set (46Ab-S153rm-f, AAACTGNNNATTAAAAAAGTCTTT (N = A/C/G/T); 46Ab-K151r, AGTGGTAATTTTCAGACCGGTCGT) (Figure 1). The expression vector pGST-Cry46Ab-S1 [3] was used as the DNA template. Upon self-ligation of the PCR-amplified DNA fragments, the ligate was transformed into *Escherichia coli* BL21.

### 2.2. Primary Screening of Mpp46Ab S^153^ Random Mutants

The primary screening of Mpp46Ab mutants was performed as previously described [6]. The *E. coli* colonies generated by mutagenesis were precultured at 37 °C overnight (O/N) in 0.5 mL of LB medium containing 100 μg/mL of ampicillin. The O/N culture (0.1 mL) was then transferred to 0.5 mL of freshly prepared LB medium (containing 100 μg/mL of ampicillin and 0.2 mM of isopropyl-β-D-thiogalactopyranoside [IPTG]) and cultured at 30 °C for an additional 4 h to induce Mpp46Ab mutant expression. The expression of Mpp46Ab did not seem to affect the *E. coli* culture. The OD_600_ for the culture of all mutants usually reached about 1.2 after incubation.

The mosquito larvae used for primary screening were reared from eggs kindly provided by the Research and Development Laboratory, Dainihon Jochugiku Co., Ltd. (Osaka, Japan). The bioassay was performed in a 96-well microtiter plate, with one mosquito larva (3rd instar) per well. For the bioassay, 10 μL of *E. coli* culture expressing each Mpp46Ab mutant was taken and administered to the mosquito larvae in the wells as a diet. Larval mortality was monitored 48 h after dosing. Eight larvae were used to evaluate the toxicity of each mutant clone, and toxicity resulting in >25% mortality (more than 3 of 8 larvae) was considered significant. The mutation in a clone showing significant toxicity was identified by DNA sequencing analysis.

### 2.3. Preparation of Mpp46Ab and Cry4Aa Toxin Proteins

Wild-type and mutant Mpp46Abs were expressed as glutathione *S*-transferase (GST) fusion proteins in *E. coli*. The *E. coli* cells were cultured at 37 °C in Terrific broth containing ampicillin (100 μg/mL) until the OD_600_ reached 0.5–0.7, and the expression of GST-Mpp46Abs was induced by incubating the cells at 30 °C for another 4 h in medium containing 0.1 mM of IPTG. GST-Mpp46Abs were purified using glutathione-Sepharose 4B resin (GE Healthcare Bio-Science AB, Uppsala, Sweden) according to the manufacturer’s instructions. For electrophysiologic analyses, the GST-Mpp46Abs were activated via passage through an immobilized trypsin column prepared as previously described [6].

Similarly, Cry4Aa was expressed as a GST fusion, as previously described [16]. The expression of GST-Cry4Aa was induced by incubation at 20 °C for an additional 4 h in 0.1 mM of IPTG. The protein concentration was estimated using a protein assay dye reagent (Bio-Rad Laboratories, Inc., Hercules, CA, USA) with bovine serum albumin as the standard. Purified proteins were analyzed by sodium dodecyl sulfate–polyacrylamide gel electrophoresis (SDS-PAGE), followed by the visualization of protein bands by staining with Coomassie brilliant blue (CBB stain one, Nacalai Tesque, Inc., Kyoto, Japan).

### 2.4. Measurement of Mosquito-Larvicidal Activity

The mosquito-larvicidal activity of GST-fused toxin proteins was determined as previously described [6]. Purified toxin proteins (20 μg) were adsorbed onto 1 mg of latex beads (0.8 μm diameter, Sigma-Aldrich Corp., St. Louis, MO, USA) for 1 h at room temperature and then administered as a diet to *C. pipiens* mosquito larvae. Bioassays were performed in a 96-well microtiter plate with a single larva (3rd instar) per well, and 24 larvae were used for each concentration of toxin in an assay. Mortality was recorded 48 h after toxin administration, and the 50% lethal concentration (LC_50_) was determined using PROBIT analysis [17]. A minimum of four replicates of each bioassay were performed in this study.

To evaluate synergistic toxicity, purified GST-Cry4Aa was mixed with each GST-Mpp46Ab mutant at a 1:1 (*w*/*w*) ratio and then adsorbed onto latex beads. After determining the LC_50_ values 48 h after the administration of single and mixed toxins, the synergism factor (SF = LC_50_ expected/LC_50_ observed) was calculated, as previously described [18,19]. In the above formula, “LC_50_ expected” was defined as the LC_50_ value of the model without interactive effects between Cry4Aa and the Mpp46Ab mutants, calculated on the basis of the LC_50_ value of individual toxins and their relative proportions in the toxin mixture [19].

### 2.5. Electrophysiologic Analysis

Electrophysiologic analyses of Mpp46Ab mutant pores were performed using agarose gel-supported lipid bilayers constructed as described previously [20]. Briefly, the electrophysiological apparatus consisted of two chambers (*cis* and *trans*), such that the voltage in the *cis* chamber was connected to a patch-clamp amplifier via a membrane potential defined by an Ag/AgCl electrode. The *cis* chamber consisted of a generic gel-loading tip filled with recording solution containing 1% agarose. The agarose was allowed to solidify, with a small amount protruding from the tip. By contrast, the *trans* chamber consisted of a generic 0.6 mL microtube, which contained recording solution with the activated Mpp46Ab mutant toxin (2.0 μg/mL) and was overlaid with phosphatidylcholine solution (40 mg/mL in *n*-decane). To generate lipid bilayers, the *cis* chamber was inserted into a lipid solution in the *trans* chamber, and the protruding portion of 1% agarose gel was placed at the interface between the lipid solution and recording solution.

The single-channel conductance of the toxin pores was determined using a symmetrical recording solution containing 150 mM of KCl and 10 mM of Tris-HCl [pH 8.0] in both chambers. To analyze the anion-cation selectivity of the toxin pores, channel currents were recorded in the presence of a 4-fold KCl gradient across the lipid bilayer (150 mM of KCl and 10 mM of Tris-HCl [pH 8.0] in the *cis* chamber, 600 mM of KCl and 10 mM of Tris-HCl [pH 8.0] in the *trans* chamber). To analyze the cation preference (K^+^, Na^+^, or Ca^2+^), the recording solution in the *trans* chamber was replaced with a salt solution (150 mM NaCl and 10 mM Tris-HCl [pH 8.0] or 75 mM CaCl_2_ and 10 mM Tris-HCl [pH 8.0]).

Data were analyzed using pClamp software, ver. 11.1 (Axon Instruments, Roster City, CA, USA). The recorded currents were plotted against the corresponding applied voltage to generate current–voltage relationship graphs. Channel conductance was determined from the slope of the current–voltage relationship. The zero-current reversal potential (*V*_R_) was obtained as the V-intercept of the current–voltage relationship line and then corrected by the value of the junction potential, as previously described [16]. The ion permeability ratio for the toxin pores was calculated using the Goldman–Hodgkin–Kats equation with the *V*_R_, as previously reported [21]. Statistical significance was evaluated using the Student’s *t* test.

## 3. Results

### 3.1. Mpp46Ab Mutant Library

**Figure 2:**
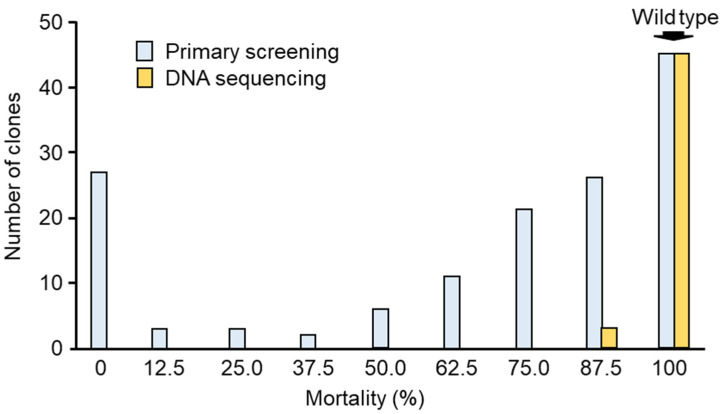
Summary of the primary screening. Of 144 randomly selected clones, 111 clones were positive (mortality > 25%). Forty-eight clones with higher toxicity were further selected for DNA sequencing analysis.

After site-directed mutagenesis, 144 clones randomly selected from the resulting *E. coli* colonies were subjected to primary screening using *C. pipiens* mosquito larvae. *E. coli* cells expressing Mpp46Ab mutants were administered directly to mosquito larvae as a diet, and the mortality rate was recorded 48 h after administration. The expression level of the Mpp46Ab mutants in *E. coli* cells was not evaluated in order to increase screening efficiency. Clones that produced a mortality rate of >25% (more than 3 of 8 larvae) were considered positive with significant toxicity. Using this approach, 111 of the 144 selected clones (77%) were positive, with 45 clones causing 100% mortality, including wild-type GST-Mpp46Ab (Figure 2). Forty-eight clones, primarily those exhibiting stronger toxicity, were selected and analyzed by DNA sequencing to determine the codon encoding amino acid residue 153 (Figure 2).

DNA sequencing analysis revealed that the Mpp46Ab mutant library consisted of 11 types of mutants, including wild-type Mpp46Ab (Table 1). Interestingly, in most of the mutants, residue S^153^ was replaced with a more hydrophobic residue (Table 1). The mutant most frequently isolated in the library was S153G (11 clones), followed by S153V (8 clones), wild-type Mpp46Ab (7 clones), and S152L (6 clones). Neither stop codons nor unexpected mutations were found in the analyzed clones. The nucleotide sequences of the mutant *mpp46Ab* genes completely matched the sequence of the wild-type gene (*cry46Ab-S1*, [3]), except for the triplet code for residue 153.

### 3.2. Mosquito-Larvicidal Activity of Mpp46Ab Mutants

**Figure 3:**
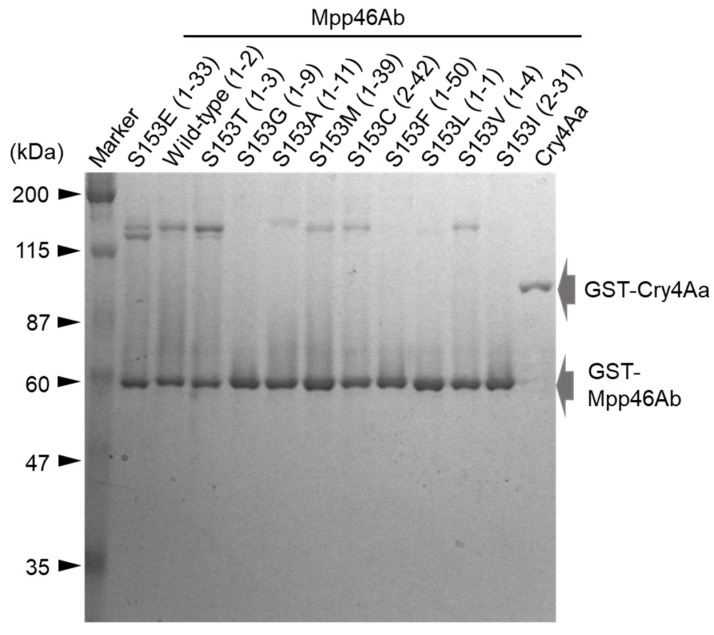
Electrophoretic analysis of the recombinant toxin proteins. GST-fused Mpp46Ab mutants and Cry4Aa were purified using glutathione beads. The purified toxin proteins were analyzed using 10% SDS-PAGE. A total of 500 ng of purified protein was loaded in each lane. Representative clones of the Mpp46Ab mutants are shown in parentheses.

Mosquito-larvicidal activity was evaluated using GST-fused Mpp46Ab mutants prepared from the representative clones (Table 2). SDS-PAGE analysis of the purified proteins revealed that the mutant GST-Mpp46Abs were approximately 60 kDa in mass (Figure 3). This was similar to the expected mass (59.3 kDa), and no apparent difference in size was observed between the wild-type and mutant toxins. In addition, several protein bands of higher molecular mass, indicative of oligomerization, were observed in the wild-type and several mutant toxins (Figure 3). The presence of similar higher-molecular-weight molecules has been reported in previous studies, but the involvement of these proteins in mediating insecticidal activity remains unknown [3,6]. LC_50_ values at 48 h after administration were calculated for the purified proteins based on the results of the *C. pipiens* mosquito larvae bioassay.

### 3.3. Mosquito-Larvicidal Synergy Between the Mpp46Ab Mutants and Cry4Aa

Synergistic toxicity with Cry4Aa is a notable feature of Mpp46Ab. Therefore, we investigated the effect of replacing residue S^153^ of Mpp46Ab on synergism with Cry4Aa. Each S^153^ mutant was mixed with GST-Cry4Aa at a ratio of 1:1 (*w*/*w*), and the resulting toxin mixture was administered to *C. pipiens* mosquito larvae. Mortality was monitored 48 h after administration, and the LC_50_ value was calculated for each mixture. The mixture containing wild-type Mpp46Ab exhibited significantly greater toxicity than the individual toxins, with an LC_50_ value (95% confidence interval) of 0.091 (0.088–0.095) μg/mL (Table 3). The SF value was calculated to be 6.3, comparable to the previously reported SF value (5.05) [3]. Similarly, most of the Mpp46Ab mutants showed synergistic toxicity with Cry4Aa, with SF values for the toxin mixtures ranging from 4.6 to 7.9 (Table 3).

In contrast to the other mutants, Mpp46Ab mutants S153F and S153L did not exhibit significant synergism. The LC_50_ values (95% confidence interval) for toxin mixtures containing the S153F and S153L mutants were low, at 0.302 (0.284–0.321) μg/mL and 0.315 (0.300–0.331) μg/mL, respectively, with corresponding calculated SF values of 2.1 and 1.9, respectively (Table 3). The S153I mutant exhibiting increased toxicity represented another exception. Contrary to our expectation, the LC_50_ value (95% confidence interval) for the toxin mixture containing S153I was 0.096 (0.090–0.102) μg/mL, similar to that of the toxin mixture containing wild-type Mpp46Ab (Table 3). This also resulted in a low SF value (2.6) for the toxin mixture containing S153I (Table 3). The decline in the SF value suggested that these mutations adversely impacted some aspect of the synergism between Mpp46Ab and Cry4Aa.

### 3.4. Single-Channel Analysis of Toxin Pores Formed by Selected Mpp46Ab Mutants

**Figure 4:**
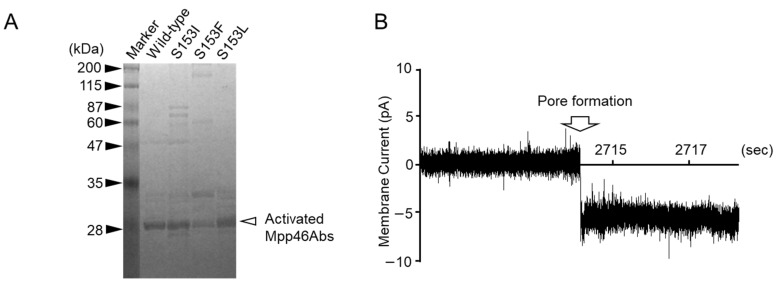
Single-channel analysis of Mpp46Ab channel pore conductance: (**A**) 10% SDS-PAGE analysis of activated Mpp46Ab toxins; (**B**) typical current trace recorded for the formation of wild-type Mpp46Ab channel pores in an artificial lipid bilayer. Channel pore formation was facilitated by applying a holding potential of −70 mV across the lipid bilayer.

As described above in Section 3.2 and Section 3.3, the Mpp46Ab mutants S153F, S153L, and S153I showed clear differences in toxicity and/or synergism with Cry4Aa compared to the wild-type toxin. As S^153^ is located within the transmembrane β-hairpin, these differences were thought to be caused by a mutation-induced change in ion permeability through the toxin pores. To evaluate this hypothesis, the ion permeability of toxin pores formed by Mpp46Abs was analyzed using an electrophysiologic method with artificial lipid bilayers. Wild-type and mutant Mpp46Abs expressed as GST fusion proteins were activated as previously described [3]. SDS-PAGE analysis revealed a 29-kDa protein for all trypsin-activated Mpp46Abs (Figure 4A). Several protein bands of higher molecular mass were attributed to toxin oligomers. The size and pattern of the Mpp46Ab oligomers varied among the mutants but did not correlate with their toxicity (Figure 4, Table 2). In general, oligomerization is considered essential for the pore formation of Mpp toxins and subsequent cell death [23], but the involvement of the oligomers observed in this study is unknown. Activated Mpp46Ab solutions were desalted using an ultrafiltration device and then subjected to measurement. The channel current through the Mpp46Ab pores was measured under symmetric buffer conditions (150 mM KCl, 10 mM Tris-HCl [pH 8.0]). Pore formation was facilitated by applying a holding potential of −70 mV across the lipid bilayer. Measurements were repeated more than three times using independently prepared activated Mpp46Abs.

A current spike indicative of Mpp46Ab pore formation was typically observed between 40 and 50 min after preparation of the agarose gel-supported lipid bilayer (Figure 4B). Mpp46Ab-induced pores in the lipid bilayer appeared to remain in a stable open state for at least several minutes, as previously reported [20].

**Figure 5:**
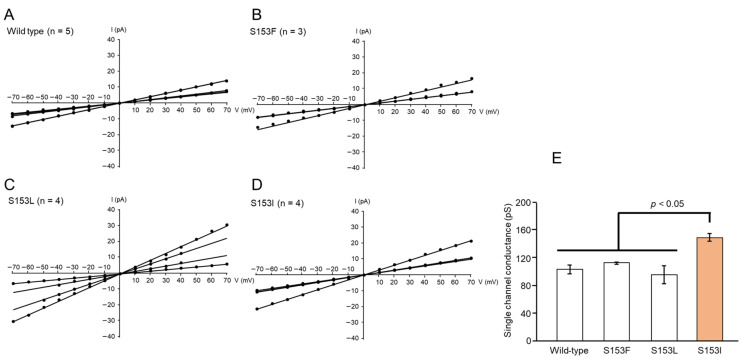
Current–voltage relationship of Mpp46Ab channel pores: (**A**) wild-type, (**B**) S153F, (**C**) S153L, (**D**) S153I, and (**E**) comparison of single-channel conductance. Channel conductance was determined from the slope of the line (Panel (**A**–**D**)). Statistical significance was evaluated using the Student’s *t* test.

Membrane currents were recorded between −70 and +70 mV and plotted against the corresponding applied voltage to generate current–voltage relationships. The current–voltage relationship for the toxin pores formed by wild-type Mpp46Ab was linear, but lines with two different conductance levels (approximately 100 and 200 pS) were also observed (Figure 5A). The lines with higher conductance (200 pS) were assumed to be generated by the presence of two toxin pores in the lipid bilayer; therefore, the single-channel conductance of wild-type Mpp46Ab pores in symmetric buffer containing 150 mM KCl was determined to be 103.9 ± 6.4 pS. This value was similar to that reported previously [6,20].

Interestingly, the single-channel conductance appeared to be correlated with the toxicity of the selected Mpp46Ab mutants (Figure 5B–D). For example, the toxicity of the S153F and S153L mutants was similar to that of wild-type Mpp46Ab, and the single-channel conductance of these mutants was also similar to that of wild-type Mpp46Ab, at 113.3 ± 0.9 and 96.6 ± 13.0 pS, respectively (Figure 5E). The single-channel conductance of S153I exhibiting increased toxicity was 150.1 ± 5.6 pS, significantly higher than that of wild-type Mpp46Ab (Figure 5E). By contrast, no correlation was observed between single-channel conductance and synergistic toxicity with Cry4Aa.

### 3.5. Anion-Cation Selectivity of Toxin Pores Formed by Selected Mpp46Ab Mutants

An apparent correlation between the insecticidal activity and anion-cation selectivity of the toxin pores has been reported for Mpp46Ab and Cry4Aa toxins [6,24]. Therefore, we investigated the anion-cation selectivity of the toxin pores to evaluate the possibility of such a correlation for the Mpp46Ab mutants constructed in this study. Membrane currents were measured in the presence of a 4-fold gradient of KCl across the lipid bilayer (150 mM KCl in the *cis* chamber, 600 mM KCl in the *trans* chamber). Recordings were repeated four times independently.

**Figure 6:**
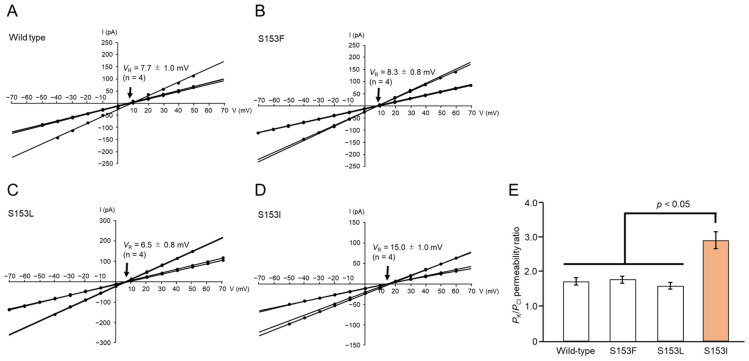
Anion-cation selectivity of Mpp46Ab channel pores. Channel currents were recorded in the presence of a 4-fold KCl gradient across the lipid bilayer. The experiment was repeated four times independently. (**A**) Current–voltage relationship of wild-type Mpp46Ab channel pores. (**B**) S153F. (**C**) S153L. (**D**) S153I. Mean (standard deviation) *V*_R_ was determined from each fitted line. (**E**) Comparison of *P*_K_/*P*_Cl_ permeability ratios. *P*_K_/*P*_Cl_ permeability ratios were calculated from the *V*_R_ values obtained in panels (**A**–**D**). Statistical significance was evaluated using the Student’s *t* test.

As observed in the single-channel conductance analysis above, the current–voltage relationship was linear for all Mpp46Ab mutants, but the *V*_R_ value (V-intercept of the lines) varied among the mutants (Figure 6A–D). The *V*_R_ value for the pores formed by wild-type Mpp46Ab was 7.7 ± 1.0 mV (Figure 6A). After correction for the junction potential (0.4 mV), the *P*_K_/*P*_Cl_ permeability ratio was calculated to be 1.71 ± 0.11, similar to the previously reported value (1.86) [6]. As expected, the analysis of selected Mpp46Ab mutants revealed an apparent correlation between the *P*_K_/*P*_Cl_ permeability ratio and toxicity. The *P*_K_/*P*_Cl_ permeability ratios for pores formed by the S153F and S153L mutants were 1.78 ± 0.10 and 1.58 ± 0.09, respectively, similar to those of wild-type Mpp46Ab (Figure 6E). By contrast, the *P*_K_/*P*_Cl_ permeability ratio for the highly active S153I mutant was 2.90 ± 0.25, significantly higher than that of wild-type Mpp46Ab (Figure 6E). No correlation was observed between the *P*_K_/*P*_Cl_ permeability ratio and synergistic toxicity with Cry4Aa.

### 3.6. Cation Preference of Toxin Pores Formed by Selected Mpp46Ab Mutants

Although the correlation with toxicity remains unknown, it has been shown that Mpp46Ab pores exhibit an apparent preference for cation permeation (K^+^ > Na^+^, K^+^ > Ca^2+^, and Ca^2+^ > Na^+^) [6]. To determine whether cation preference is involved in toxicity and/or synergism with Cry4Aa in this study, the cation preference of mutant Mpp46Ab pores was analyzed.

**Figure 7:**
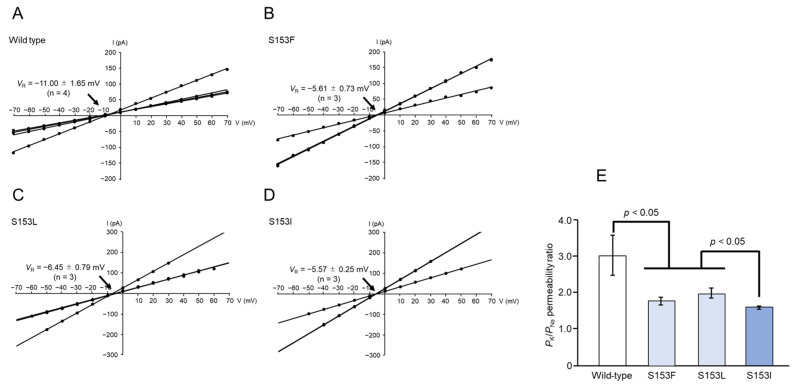
Cation selectivity (K^+^ versus Na^+^) of Mpp46Ab channel pores. Channel current was recorded under asymmetric buffer conditions (150 mM KCl in the *cis* chamber, 150 mM NaCl in the *trans* chamber). (**A**) Current–voltage relationship of wild-type Mpp46Ab channel pores. (**B**) S153F. (**C**) S153L. (**D**) S153I. Mean (standard deviation) *V*_R_ was determined from each fitted line. (**E**) Comparison of *P*_K_/*P*_Na_ values. Permeability ratios were calculated from *V*_R_ values obtained in panels (**A**–**D**). Statistical significance was evaluated using the Student’s *t* test.

Under asymmetric buffer conditions (150 mM KCl in the *cis* chamber, 150 mM NaCl in the *trans* chamber), the *V*_R_ value for wild-type Mpp46Ab pores was −11.00 ± 1.65 mV (Figure 7A). After correcting the *V*_R_ value for the junction potential (−3.0 mV), the calculated *P*_K_/*P*_Na_ permeability ratio was 3.05 ± 0.54 (n = 4). This indicated that wild-type Mpp46Ab pores preferentially allow the permeation of K^+^ over Na^+^, consistent with previous reports [6]. By contrast, the *V*_R_ values for mutant toxin pores generally exhibited a positive shift (Figure 7B–D). The resulting calculated *P*_K_/*P*_Na_ permeability ratios for the S153F, S153L, and S153I mutants were 1.80 ± 0.11 (n = 3), 2.01 ± 0.14 (n = 3), and 1.61 ± 0.03 (n = 3), respectively, which were significantly lower than that of the wild-type toxin (Figure 7E).

**Figure 8:**
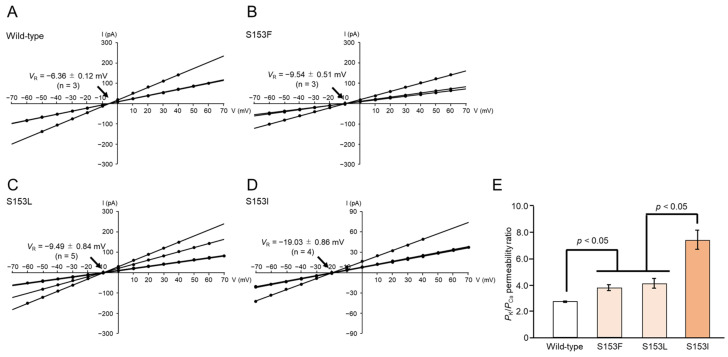
Cation selectivity (K^+^ versus Ca^2+^) of Mpp46Ab channel pores. Channel current was recorded under asymmetric buffer conditions (150 mM KCl in the *cis* chamber, 75 mM CaCl_2_ in the *trans* chamber). (**A**) Current–voltage relationship of wild-type Mpp46Ab channel pores. (**B**) S153F. (**C**) S153L. (**D**) S153I. Mean (standard deviation) *V*_R_ was determined from each fitted line. (**E**) Comparison of *P*_K_/*P*_Ca_ values. Permeability ratios were calculated from *V*_R_ values obtained in panels (**A**–**D**). Statistical significance was evaluated using the Student’s *t* test.

Similarly, under a different combination of buffer conditions (150 mM KCl in the *cis* chamber, 75 mM CaCl_2_ in the *trans* chamber), the *V*_R_ value for wild-type Mpp46Ab pores was −6.36 ± 0.12 mV (Figure 8A). After correcting the *V*_R_ value for the junction potential (−4.7 mV), the calculated *P*_K_/*P*_Ca_ permeability ratio was 2.72 ± 0.04 (n = 3). This indicated that wild-type Mpp46Ab pores preferentially allow the permeation of K^+^ over Ca^2+^, consistent with previous reports [6]. In contrast to the case of K^+^ versus Na^+^, the *V*_R_ values for the mutant toxins generally exhibited a negative shift in this analysis (Figure 8B–D). The calculated *P*_K_/*P*_Ca_ permeability ratios for the S153F, S153L, and S153I mutants were 3.79 ± 0.22 (n = 3), 4.16 ± 0.42 (n = 5), and 7.44 ± 0.73 (n = 4), respectively, significantly higher than that of the wild-type toxin (Figure 8E). Thus, the toxin pores formed by the S153F, S153L, and S153I Mpp46Ab mutants, which exhibited reduced synergy with Cry4Aa, not only showed significantly reduced *P*_K_/*P*_Na_ permeability ratios but also significantly increased *P*_K_/*P*_Ca_ permeability ratios compared to pores formed by the wild-type toxin.

## 4. Discussion

In a series of analyses used to elucidate the role of amino acid residues within the transmembrane β-hairpin, we constructed an Mpp46Ab mutant library, in which residue S^153^ was randomly replaced with other amino acids. A total of 144 clones were subjected to the primary screening using *C. pipiens* mosquito larvae, and ultimately, 11 Mpp46Ab mutants, including the wild type, were obtained (Table 1). Interestingly, in most of the Mpp46Ab mutants, S^153^ was replaced with a more hydrophobic amino acid, suggesting that increased hydrophobicity at residue 153 may impart an advantage that enhances mosquito-larvicidal activity (Table 1). Since subsequent bioassays using purified proteins revealed significant toxicity of all mutants against *C. pipiens* mosquito larvae (Table 2), the primary screening was considered successful. Given the success observed in this and a previous study [6], this primary screening approach should be useful for selecting highly active mosquito-larvicidal mutant toxins. However, mutant toxins excluded from the Mpp46Ab library may have a structural issue affecting the toxin pores and/or an issue affecting the toxin molecule itself that suppresses mosquito-larvicidal activity. It is also possible that some mutants were not selected by chance in the primary screening.

The S153I mutant with a significant increase in toxicity (Table 2) was of particular interest among the Mpp46Ab mutants listed in the library. Because residue S^153^ is located within the transmembrane β-hairpin of Mpp46Ab, we speculated that the observed changes in toxicity were caused by changes in ion permeability through the mutant toxin pores. Indeed, electrophysiologic analyses using artificial lipid bilayers revealed an apparent correlation between mosquito-larvicidal activity and the ion permeability of the toxin pores, specifically with regard to single-channel conductance and the *P*_K_/*P*_Cl_ permeability ratio (Figure 5E and Figure 6E). An increase in the *P*_K_/*P*_Cl_ permeability ratio was also reported for K155E and K155I Mpp46Ab mutants, which exhibited increased toxicity [6]. The formation of highly ion-permeable and highly cation-selective pores, as observed in S153I mutants, is thought to increase the influx of cations and water into mosquito larvae target cells, thereby facilitating the development of osmotic shock and the eventual death of the larvae. In light of these observations, polar residues such as S^153^ and K^155^ within the transmembrane β-hairpin may be good targets for improving toxicity via mutagenesis.

We also evaluated the effect of replacing Mpp46Ab residue S^153^ on synergistic toxicity with Cry4Aa. Synergistic toxicity has frequently been reported in many combinations of *B. thuringiensis* insecticidal proteins, in addition to the combination of Cry4Aa and Mpp46Ab. For example, Cyt1Aa from Bti shows low toxicity against mosquito larvae but, through synergism, exhibits enhanced toxicity in conjunction with other Cry toxins [25,26,27,28,29]. The Bti toxins Cry4Aa, Cry4Ba, and Cry11Aa share a similar three-domain architecture, and the combination of Cry4Aa and Cry4Ba or Cry11Aa, but not Cry4Ba and Cry11Aa, shows synergistic toxicity against mosquito larvae [30]. Synergistic toxicity against the polyphagous caterpillar *Spodoptera frugiperda* was reported for mixtures containing selected Cry1, Cry2, and Vip3 insecticidal proteins [31]. We also previously observed synergistic toxicity between Cry4Aa and Cry11Ba against *C. pipiens* mosquito larvae, in addition to the combination of Mpp46Ab and Cry4Aa [18]. Another study showed that Cyt1Aa acts as a surrogate receptor for Cry11Aa in the midgut of mosquito larvae [26]. However, it is not yet clear how other toxins interact with each other to exhibit synergistic activity. It is generally believed that synergistic toxicity occurs when multiple toxins with different modes of action are administered together, but the differences that lead to synergy have not been identified.

Notably, we identified potential indicators that may correlate with the synergistic toxicity between Mpp46Ab and Cry4Aa observed in this study. Electrophysiologic analyses of mutant toxin pores revealed that mutants exhibiting reduced synergy (S153F, S153L, and S153I) form toxin pores with a significantly reduced *P*_K_/*P*_Na_ permeability ratio and a significantly increased *P*_K_/*P*_Ca_ permeability ratio compared to the wild-type toxin (Figure 7E and Figure 8E). A comparison of the ion selectivity of Mpp46Ab and Cry4Aa toxin pores revealed that both are cation selective, but there are apparent differences in the cations that preferentially pass through the pores [6,16]. The reported *P*_K_/*P*_Na_ permeability ratio for Cry4Aa pores is approximately 0.7 [16], which is significantly lower than that of Mpp46Ab pores determined in this study (3.05 ± 0.54). Therefore, the reduced *P*_K_/*P*_Na_ permeability ratios for pores of the S153F, S153L, and S153I Mpp46Ab mutants (ranging from 1.61 to 2.01) can be explained as being closer to that of Cry4Aa. Similarly, the reported *P*_K_/*P*_Ca_ permeability ratio for Cry4Aa pores is approximately 3.4 [16], apparently higher than that of Mpp46Ab pores determined in this study (2.72 ± 0.04). Thus, the increased *P*_K_/*P*_Ca_ permeability ratios of the mutant toxins (ranging from 3.79 to 7.44) can also be explained in a similar way. Pore formation by *B. thuringiensis* insecticidal proteins is generally thought to facilitate the influx of water along with ions, leading to the swelling and eventual lysis of target cells [32]. However, the influx of specific ions into target cells may trigger a cascade of physiologic processes that ultimately lead to programmed death. Therefore, it is reasonable to speculate that differences in the ion permeability of toxin pores may result in different effects on target insects.

At this time, we do not know how the permeability of toxin pores, specifically the *P*_K_/*P*_Na_ and/or *P*_K_/*P*_Ca_ permeability ratios, is involved in the mechanism of synergistic toxicity. In particular, the interaction between Mpp46Abs and Cry4Aa was not evaluated in this study, leaving open the possibility that one toxin could influence pore formation by another toxin. More detailed studies using a toxin mixture are needed to fully elucidate the effect of toxin pore ion permeability on synergistic toxicity. In contrast to the present study, future studies should investigate the ion permeability of constructed Cry4Aa mutants exhibiting reduced synergy. It would also be of great interest to investigate the physiologic effects of the S153F, S153L, and S153I Mpp46Ab mutant toxins in susceptible cells.

## 5. Conclusions

We constructed Mpp46Ab mutants in which residue S^153^ was randomly replaced with other amino acids. A total of 11 Mpp46Ab mutants were selected using a simplified primary screening approach, and further analysis revealed that, in most of the mutants, residue S^153^ was replaced with a more hydrophobic residue. In bioassays using *C. pipiens* mosquito larvae, mutant S153I exhibited significantly greater toxicity. In addition, three Mpp46Ab mutants, S153F, S153L, and S153I, exhibited a significant reduction in synergistic toxicity with Cry4Aa toxin. Interestingly, electrophysiologic analyses of the mutant toxin pores revealed a significant increase in both single-channel conductance and *P*_K_/*P*_Cl_ permeability ratio for the S153I mutant. By contrast, a significant reduction in the *P*_K_/*P*_Na_ permeability ratio and a significant increase in the *P*_K_/*P*_Ca_ permeability ratio were observed for the S153F, S153L, and S153I mutants. Our results suggest that pore formation is central to the mode of action of Mpp46Ab; therefore, the ion permeability of Mpp46Ab pores affects both its own toxicity and the synergistic toxicity with Cry4Aa. The ion permeability of Mpp46Ab pores can be controlled by targeting amino acid residues such as K^155^ and S^153^ within the transmembrane β-hairpin region using mutagenesis.

## Figures and Tables

**Table 1:** Summary of the Mpp46Ab S^153^ random mutant library.

Amino Acid	Hydropathy Index ^1^	Side Chain (Electrical Charge)	Number of Clones	Name
R	−4.5	Positive	0	-
K	−3.9		0	-
H	−3.2		0	-
D	−3.5	Negative	0	-
E	−3.5		1	S153E
N	−3.5	Neutral	0	-
Q	−3.5		0	-
P	−1.6		0	-
Y	−1.3		0	-
W	−0.9		0	-
S	−0.8		7	Wild type
T	−0.7		4	S153T
G	−0.4		11	S153G
A	1.8		4	S153A
M	1.9		1	S153M
C	2.5		1	S153C
F	2.8		4	S153F
L	3.8		6	S153L
V	4.2		8	S153V
I	4.5		1	S153I

^1^ Values as defined by Kyte and Doolittle [22].

**Table 2:** Mosquito-larvicidal activity of Mpp46Ab S^153^ mutants.

Toxin	Replication (n)	Mosquito-Larvicidal Activity (μg/mL)		Representative Clone
		LC_50_	95% Confidence Interval	
S153E	5	0.48	0.42–0.54	1–33
Wild type (S)	6	0.62	0.56–0.69	1–2
S153T	6	0.79	0.71–0.89	1–3
S153G	4	0.78	0.72–0.85	1–9
S153A	5	0.66	0.59–0.72	1–11
S153M	5	0.70	0.64–0.78	1–39
S153C	6	1.07	0.98–1.19	2–42
S153F	6	0.76	0.69–0.84	1–50
S153L	6	0.66	0.60–0.72	1–1
S153V	4	0.79	0.72–0.87	1–4
S153I	9	0.16	0.13–0.19	2–31
Cry4Aa	3	0.53	0.50–0.57	

**Table 3:** Synergy of mosquito-larvicidal activity between Mpp46Ab S^153^ mutants and Cry4Aa.

Mpp46Ab	LC_50_ (μg/mL)		Synergism Factor (LC_50_ Expected/LC_50_ Observed)
	Expected ^1^	Observed (95% Confidential Interval)	
S153E	0.50	0.105 (0.098–0.111)	4.8
Wild type (S)	0.57	0.091 (0.088–0.095)	6.3
S153T	0.63	0.080 (0.076–0.084)	7.9
S153G	0.63	0.136 (0.129–0.143)	4.6
S153A	0.59	0.081 (0.076–0.086)	7.3
S153M	0.60	0.086 (0.081–0.091)	7.0
S153C	0.71	0.090 (0.086–0.095)	7.9
S153F	0.62	0.302 (0.284–0.321)	2.1
S153L	0.59	0.315 (0.300–0.331)	1.9
S153V	0.63	0.088 (0.082–0.093)	7.2
S153I	0.25	0.096 (0.090–0.102)	2.6

^1^ Expected toxicity (LC_50_) was calculated as described by Tabashnik [19].

## Data Availability

Data are available from the authors upon reasonable request.
